# The Involvement of Neuron-Specific Factors in Dendritic Spinogenesis: Molecular Regulation and Association with Neurological Disorders

**DOI:** 10.1155/2016/5136286

**Published:** 2015-12-24

**Authors:** Hsiao-Tang Hu, Pu-Yun Shih, Yu-Tzu Shih, Yi-Ping Hsueh

**Affiliations:** Institute of Molecular Biology, Academia Sinica, Taipei 11529, Taiwan

## Abstract

Dendritic spines are the location of excitatory synapses in the mammalian nervous system and are neuron-specific subcellular structures essential for neural circuitry and function. Dendritic spine morphology is determined by the F-actin cytoskeleton. F-actin remodeling must coordinate with different stages of dendritic spinogenesis, starting from dendritic filopodia formation to the filopodia-spines transition and dendritic spine maturation and maintenance. Hundreds of genes, including F-actin cytoskeleton regulators, membrane proteins, adaptor proteins, and signaling molecules, are known to be involved in regulating synapse formation. Many of these genes are not neuron-specific, but how they specifically control dendritic spine formation in neurons is an intriguing question. Here, we summarize how ubiquitously expressed genes, including syndecan-2, NF1 (encoding neurofibromin protein), VCP, and CASK, and the neuron-specific gene CTTNBP2 coordinate with neurotransmission, transsynaptic signaling, and cytoskeleton rearrangement to control dendritic filopodia formation, filopodia-spines transition, and dendritic spine maturation and maintenance. The aforementioned genes have been associated with neurological disorders, such as autism spectrum disorders (ASDs), mental retardation, learning difficulty, and frontotemporal dementia. We also summarize the corresponding disorders in this report.

## 1. Introduction

The tiny protrusions emerging from dendrites known as dendritic spines are the primary subcellular locations of excitatory synapses in the mammalian central nervous system [1]. Dendritic spines are typically ~1-2 *μ*m in length and 0.5–1 *μ*m in width of the spine head, with diverse morphologies, such as mushroomlike, stubby, and thin spines. These structures are mainly supported by the F-actin cytoskeleton. Thus, F-actin cytoskeletal proteins and regulators are important factors for generating dendritic spines. Many membrane proteins and adaptor and signaling molecules are also involved in controlling dendritic spine formation and maintenance [2]. Several mechanisms have been described to form dendritic spines [3]. The most popular mechanism is that dendritic filopodia serve as precursors for dendritic spine formation. Interestingly, filopodia are ubiquitously found on various cell types. In contrast, dendritic spines are neuron-specific structures. Thus, the transition from filopodia to spines should be controlled by neuron-specific factors.

Neuron-specific factors controlling dendritic spinogenesis fall into two categories. The first group is proteins specifically expressed in neurons. The second group is neuron-specific cellular responses or processes. These proteins or responses directly or indirectly regulate F-actin rearrangement and dynamics to promote dendritic spine formation. Studies of cytoskeleton-associated cortactin-binding protein 2 (CTTNBP2) and heparan sulfate transmembrane proteoglycan (HSPG) syndecan-2 serve as examples for these two categories, respectively. CTTNBP2 is a neuron-specific cytoskeleton-associated protein and that is enriched at dendritic spines of mature neurons. Although syndecan-2 is widely expressed in many cell types, it is highly concentrated at synapses in neurons. Syndecan-2 cooperates with other proteins to trigger neurotransmission through a neuron-specific signal to induce dendritic spine formation. Genomic analyses of patients with autism spectrum disorders (ASDs) indicated that both CTTNBP2 and syndecan-2 were associated with ASDs [4, 5]. Additionally, neurofibromin, CASK, and VCP coordinate with syndecan-2 to control dendritic spinogenesis and were also associated with neurological disorders. These findings suggest that these genes are critical for neuronal function, likely through their regulation of dendritic spine formation. In this review, we will summarize the functions of these proteins in dendritic spinogenesis and use these proteins as examples to discuss how neuron-specific molecules coordinate with ubiquitously expressed proteins to generate neuron-specific signals for dendritic spine formation.

## 2. The HSPG Syndecan-2 Triggers Dendritic Spine Formation

### 2.1. Syndecan-2 Is Enriched at Dendritic Spines and Is Required for Dendritic Spine Formation

**Figure 1:**
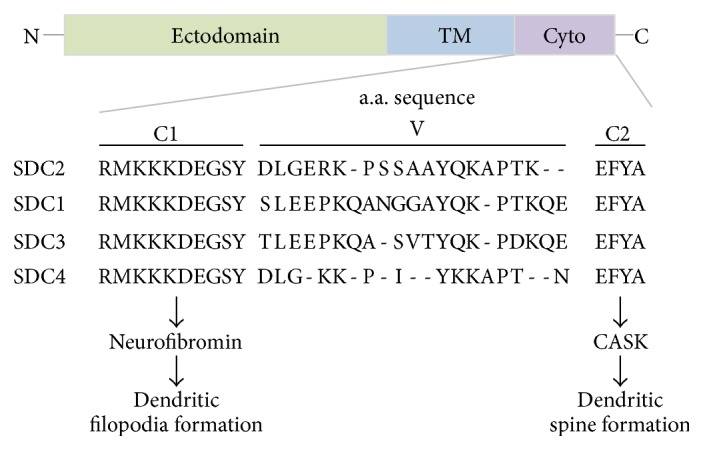
Schematic structure and amino acid sequences of syndecans. C1, conserved domain 1; C2, conserved domain 2; Cyto, cytoplasmic domain; SDC2, syndecan-2; SDC1, syndecan-1; SDC3, syndecan-3; SDC4, syndecan-4; TM, transmembrane; V, variable region.

Syndecan-2 is a type I membrane protein with a heparan sulfate modification at its ectodomain (Figure 1) [6]. In mammals, the syndecan protein family contains four members, syndecan-1, syndecan-2, syndecan-3, and syndecan-4 [7]. In rodent brains, syndecan-2 and syndecan-3 are the two major syndecans expressed in neurons with differential distribution; syndecan-2 is highly concentrated at synapses, while syndecan-3 is distributed along the axonal shaft [8]. Syndecan-2 is involved in cell-cell and cell-matrix interactions through its heparan sulfate modification. It can also bind growth factors, such as fibroblast growth factor (FGF) and epidermal cell growth factor, and it acts as a coreceptor for these growth factors [7]. Syndecan-2 is broadly and dynamically expressed in several tissues and cell types [7, 8]. During neural development, its expression gradually increases concurrent with synapse formation [8, 9]. In mature neurons, such as cultured rat hippocampal neurons at 18 days after plating* in vitro* (DIV) or later, syndecan-2 is highly enriched at dendritic spines [9, 10]. More importantly, overexpression of syndecan-2 in immature rat hippocampal cultured neurons, such as 1-2 DIV, when endogenous syndecan-2 is not yet expressed, dendritic filopodia are massively induced at 4-5 DIV and dendritic filopodia are then transformed to dendritic spines at 8-9 DIV [9, 11]. Those dendritic spines are expected to be functional, as they are adjacent to the presynaptic marker synaptophysin based on confocal microscopy [11, 12]. Syndecan-2-induced dendritic spinogenesis serves as a model to explore the mechanisms underlying the initiation of dendritic spinogenesis (namely, dendritic filopodia formation), the transition from filopodia to spines, and dendritic spine maturation and maintenance.

### 2.2. The C1 and C2 Motifs of Syndecan-2 Work Sequentially to Promote Dendritic Spinogenesis

The ectodomain of syndecan-2 heparan sulfate modification is involved in cell-cell and cell-matrix interactions [7]. Its transmembrane domain is required for homodimerization or oligomerization [13], which is critical for the protein-protein interactions of syndecan-2 [14]. The cytoplasmic domain of syndecan-2 contains only 32 amino acid residues (Figure 1). Although it is short, it is divided into three motifs, conserved domain 1 (C1), the variable region (V), and conserved domain 2 (C2). The C1 and C2 motifs are conserved among different syndecans, while the sequences of the V regions vary (Figure 1). The C1 motif is essential for syndecan-2-induced dendritic filopodia formation of rat hippocampal cultured neurons, as the syndecan-2ΔC1 mutant completely loses the ability to promote filopodia formation and spine formation at 5 as well as 9 DIV [11, 15]. The C2 is required for the dendritic filopodia-spines transition and dendritic spine maintenance [15, 16]. Expression of the C2 deletion mutant syndecan-2ΔC2 at 2 DIV promotes dendritic filopodia formation at 5 DIV. However, those filopodia are unable to transform into dendritic spine at 9 DIV [11, 15, 16]. These analyses indicate that the function of syndecan-2 in dendritic spinogenesis can be separated into two sequential steps, namely, filopodia and spine formation, which are controlled by two distinct motifs in syndecan-2.

Because both C1 and C2 motifs are short and lack recognizable enzymatic domains, syndecan-2 binding partners have been identified to determine its molecular mechanism underlying dendritic spine formation. Several direct binding partners (summarized in Table 1) have been identified for the C1 domains of syndecan-2, including neurofibromin [17] and ezrin [18]. The C2 motif directly interacts with syntenin [19], CASK [10], and synbindin [20]. Among these, the interactions between syndecan-2 and neurofibromin and CASK have been shown to be relevant in dendritic spine formation. Because the cytoplasmic tail of syndecan-2 is very short, it is unlikely that a single syndecan-2 molecule can simultaneously interact with all of its binding partners. Because the C1 and C2 motifs are involved in two sequential processes, it is likely that neurofibromin and CASK sequentially interact with syndecan-2. Alternatively, it is possible that because syndecan-2 forms at least a dimer through its transmembrane domain, different syndecan-2 molecules in dimers or oligomers separately interact with neurofibromin and CASK. This would suggest that syndecan-2, neurofibromin, and CASK form a single large complex. Further investigation, including coimmunoprecipitation experiments, is required to address this question.

### 2.3. Neurofibromin Interacts with the C1 Motif of Syndecan-2 and Promotes Syndecan-2-Induced Dendritic Filopodia Formation

**Figure 2:**
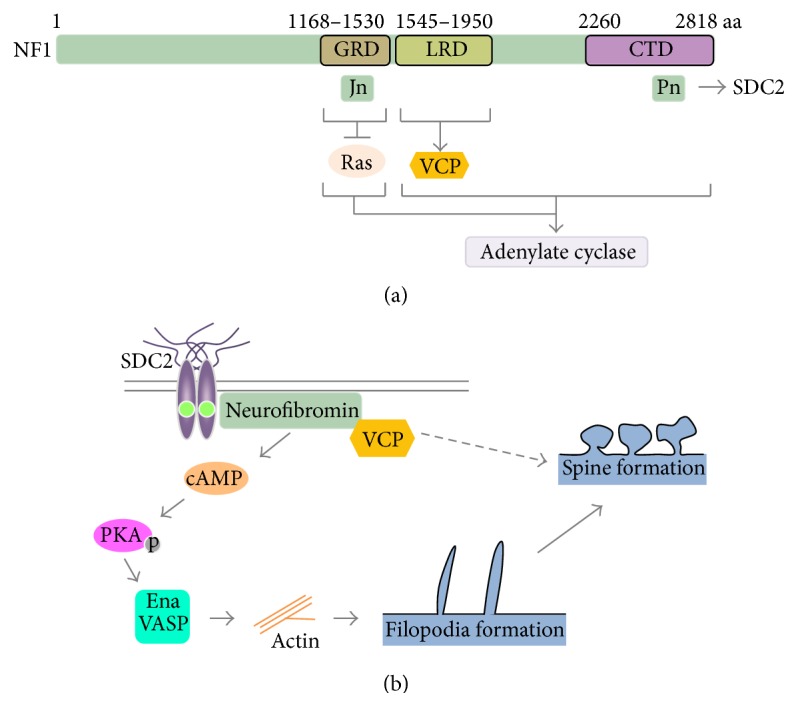
Function of neurofibromin in neurons. (a) Neurofibromin-interacting proteins. The Jn and Pn fragments interact with syndecan-2. The leucine-rich domain (LRD) binds VCP. The GAP-related domain (GRD) downregulates the Ras pathway. Both GRD and the C-terminal half of neurofibromin are involved in adenylate cyclase activity regulation. CTD, C-terminal region. (b) Neurofibromin controls dendritic filopodia and spine formation through the PKA-Ena/VASP and VCP pathways, respectively.

Neurofibromin encoded by the neurofibromatosis type I (*NF1*) gene is characterized by its RasGAP- (Ras GTPase activating protein-) related domain (GRD) (Figure 2(a)) [21–24]. Similar to syndecan-2, neurofibromin is widely expressed in different cell types, though its expression level is much higher in the nervous system [25]. NF1 is one of the most common human inherited disorders featured by changes in skin pigmentation, benign tumor growth, and learning difficulty [26, 27]. Neurofibromin suppresses tumor growth through its ability to downregulate the RAS pathway [28]. In addition to its RAS activity, neurofibromin can increase cAMP concentration by activating adenylate cyclase [29]. Although the molecular mechanisms are less clear, the GRD and C-terminal region of neurofibromin are required for cAMP pathway activation (Figure 2(a)) [30]. Both Gs-dependent and Gs-independent pathways are involved in neurofibromin-regulated adenylate cyclase activation [30]. The cAMP pathway has been shown to be involved in learning and memory in* Drosophila* [31] and dendritic spine formation in the mammalian nervous system [11].

In a yeast two-hybrid screen using different fragments of neurofibromin as baits, syndecan-2 was identified as a neurofibromin binding partner [17]. Notably, neurofibromin has two independent interacting domains for the C1 motif of syndecan-2. One is the Jn fragment corresponding to amino acid residues 1357–1473 in the GRD of human neurofibromin; the other is the Pn fragment containing amino acid residues 2619–2719 (Figure 2(a)) [17]. The Jn and Pn compete for binding to the C1 motif of syndecan-2. In addition to biochemical studies demonstrating the direct interaction between syndecan-2 and neurofibromin, fluorescence immunostaining further demonstrated the colocalization of syndecan-2 and neurofibromin at synapses in cultured hippocampal neurons [17]. Moreover, both Nf1 knockdown and haploinsufficiency reduce the density of dendritic spines in both rat hippocampal and mouse cortical cultured neurons and in brains [11, 32], consistent with a function of neurofibromin in regulating dendritic spine formation.

The next question is how the syndecan-2-neurofibromin complex regulates dendritic spine formation. One study examined syndecan-2 downstream signaling for triggering filopodia formation. Using a panel of inhibitors to suppress various kinase activities, protein kinase A (PKA) was identified to be required for syndecan-2-induced filopodia formation [11]. Combined with the analysis using different motif deletion mutants of syndecan-2, we found that the C1 motif of syndecan-2 is essential for PKA-dependent filopodia formation [11]. Because neurofibromin interacts with the C1 motif and also activates the cAMP pathway, cultured hippocampal neurons were then used to investigate whether neurofibromin mediates syndecan-2-induced filopodia formation. Both Nf1 knockdown and Jn fragment expression, which acts as a dominant-negative to disrupt the interaction between endogenous neurofibromin and syndecan-2, suppress syndecan-2-induced dendritic filopodia formation of rat hippocampal cultured neurons at 5 DIV [11]. Thus, neurofibromin mediates the signal from syndecan-2 to the cAMP pathway to initiate dendritic spinogenesis.

Because filopodia are supported by F-actin bundles, the syndecan-2-neurofibromin-cAMP pathway has to induce F-actin polymerization and bundle formation to promote dendritic filopodia formation. The Ena (Enabled)/VASP (Vasodilator-Stimulated Phosphoprotein) protein family is a group of F-actin regulators that initiate actin polymerization and bundling [33]. PKA phosphorylation promotes Ena/VASP protein activity to regulate the F-actin cytoskeleton [34]. Upon syndecan-2 overexpression, Ena/VASP phosphorylation increases, consistent with cAMP pathway activation. Moreover, disruption of Ena/VASP activity impairs syndecan-2-induced dendritic filopodia formation [11]. In summary, these studies indicate that syndecan-2 overexpression enhances the ability of neurofibromin to activate the PKA pathway, which then induces the Ena/VASP activity to promote F-actin bundling and filopodia formation.

Although the PKA pathway is required for dendritic filopodia formation, increased intracellular cAMP concentrations alone cannot induce dendritic filopodia formation [11], suggesting that other factor(s) are involved. From an immunoprecipitation-mass spectrometry study, valosin-containing protein (VCP, also known as P97) was identified as a neurofibromin-binding protein [32]. The entire D1 and D2 ATPase domains of VCP are required for the interaction with the leucine-rich domain (LRD) of neurofibromin [32]. VCP is a causative gene of inclusion body myopathy associated with Paget's disease of bone and frontotemporal dementia (IBMPFD) [35]. IBMPFD patients frequently suffer from dementia. In addition, VCP mutations are associated with ASDs and amyotrophic lateral sclerosis [36, 37]. These evidences suggest that VCP mutations impair brain function. A combination of human genetic studies, mouse genetic models, and cultured hippocampal and cortical neurons have indicated that neurofibromin interacts with VCP and guides VCP to promote dendritic spinogenesis [32]. The roles of VCP and neurofibromin in dendritic spine formation may account for the neural phenotypes in patients with mutations in the NF1 and VCP genes. However, it is still unclear how VCP regulates dendritic spine formation. To fully address the molecular regulation of neurofibromin and VCP in dendritic spinogenesis, further studies are required.

The function of the syndecan-2-neurofibromin interaction in dendritic spine formation is summarized in Figure 2(b).

### 2.4. CASK and Syndecan-2 Interactions Regulate Dendritic Spine Maturation

CASK is a ubiquitously expressed gene and is critical for brain development and function [38]. Mutations in the human CASK gene result in X-linked mental retardation and microcephaly with pontine and cerebellar hypoplasia [39–43]. CASK belongs to the membrane-associated guanylate kinase (MAGUK) family and functions as a scaffold protein to interact with more than two dozen cellular proteins [44]. It is widely distributed in neurons, including synapses, dendrites, axons, and soma [10]. At synapses, it localizes to both pre- and postsynaptic sites [10]. In mouse pontine explants and rat hippocampal cultured neurons, CASK knockdown impairs synapse formation at the pre- and postsynapse, respectively [16, 45]. At presynaptic sites, it binds the membrane protein neurexin and other scaffold proteins, such as Mint1, mLin7, and liprin, to control presynaptic button formation [45–48]. CASK uses its PDZ domain at the postsynaptic site to interact with the C2 motif of syndecan-2 [10]. In cultured hippocampal neurons, expression of the PDZ alone of CASK or the C-terminal tail of syndecan-2 that disrupts the interaction between endogenous CASK and syndecan-2 reduces dendritic spine density, narrows spine heads, and shortens spine length at 18 DIV, suggesting that the CASK-syndecan-2 interaction is critical for dendritic spine formation [16].

**Figure 3:**
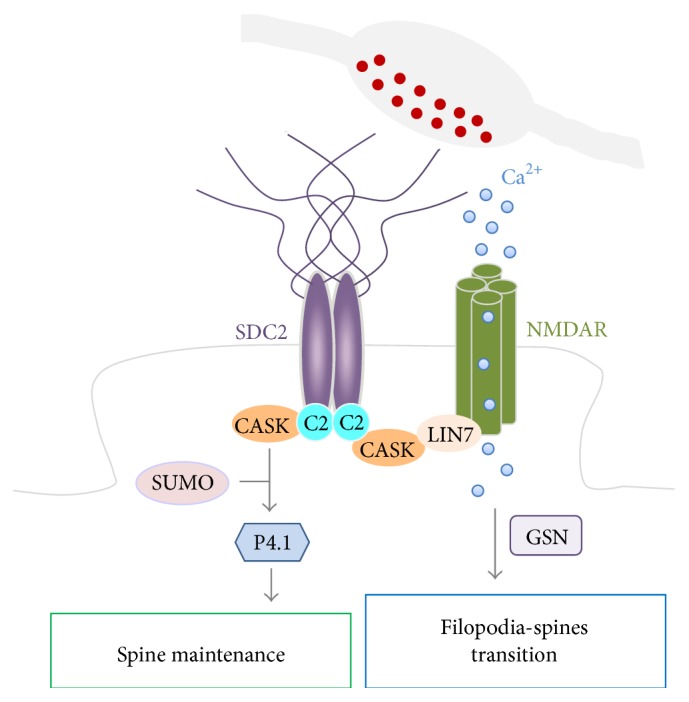
Syndecan-2 coordinates with calcium influx to control dendritic spine formation and maturation. Syndecan-2 links the CASK-mLIN7-NMDAR complex through its C2 motif and directs this complex to target to filopodial tips. It increases the accessibility of postsynaptic filopodia to presynaptic stimulation, which is critical for calcium influx to induce the filopodia-spines transition. In addition to linking mLIN7 and NMDAR, CASK interacts with the protein 4.1-F-actin cytoskeleton. This interaction provides a physical link between the membrane and cytoskeleton to stabilize the dendritic spine structure. GSN, gelsolin.

To investigate whether CASK is involved in dendritic spinogenesis initiation or dendritic spine stabilization, a time course study using a knockdown approach in cultured hippocampal neurons has been performed [16]. The time window of 15–18 DIV covering the initiation toward maturation of dendritic spinogenesis was used for analysis. At 15 DIV, wild-type dendritic spines are immature, long, and thin, and they are present at a low density. As they mature at 18 DIV, dendritic spine density increases, spine length decreases, and spine width increases. Compared to control neurons, CASK knockdown does not affect spine density, length, or size at 15 DIV, suggesting that CASK is not critical for dendritic spinogenesis initiation. At 18 DIV, CASK knockdown induces dendritic spines withdraw and the spine heads fail to enlarge. The spine density is decreased compared to control neurons [16]. The data indicate that CASK is important for dendritic spine maturation, likely by linking the membrane protein syndecan-2 to the F-actin cytoskeleton via protein 4.1 to stabilize dendritic spines (Figure 3) [16].

### 2.5. Neurotransmission-Induced Calcium Influx Is Critical for the Syndecan-2-Induced Filopodia-Spines Transition

In human embryonic kidney HEK293 cells, syndecan-2 overexpression induced numerous filopodia on the cell surface [11]. However, these filopodia cannot mature into spines. Because neurofibromin and CASK are also expressed in HEK293 cells, the aforementioned studies cannot explain why syndecan-2-induced dendritic spines are only present in neurons. A neuron-specific factor must be present to control dendritic spine formation. Because neurotransmission is a neuron-specific event and because dendritic filopodia are able to receive neurotransmission signals from presynaptic buttons [49], neurotransmission seems a likely factor that triggers the filopodia-spines transformation in a neuron-specific manner. Indeed, EGTA treatment to chelate extracellular calcium or AP5 treatment to block NMDAR activity, a major neurotransmitter gated calcium channel, impairs the endogenous filopodia-spines transition at 15–17 DIV and syndecan-2-induced filopodia-spines transition at 5–9 DIV [15]. In syndecan-2-overexpressing neurons, intracellular calcium concentration is increased compared to control neurons at 5 DIV. This increase is due to NMDAR-regulated calcium influx because AP5 treatment effectively reduced the intracellular calcium concentration induced by syndecan-2 [15]. The C2 motif of syndecan-2 is required for syndecan-2 overexpression-induced calcium influx [15], suggesting that the interaction with CASK is involved in calcium influx. Previous studies have shown that CASK interacts with mLIN7 via the L27 domains in both proteins [50–52] and that mLIN7 interacts with the C-terminal tail of NMDAR subunit 2b (NMDAR2b) through its PDZ domain [53]. Thus, the CASK-mLIN7 complex links NMDAR to syndecan-2. The interaction between syndecan-2, CASK, mLIN7, and NMDAR2b facilitates NMDAR localization to the tips of dendritic filopodia, where NMDAR may be activated by presynaptic stimulation, namely, glutamate, and induce calcium influx. Disruption of the syndecan-2, CASK, mLIN7, and NMDAR complex by overexpressing the interacting domains impairs NMDAR filopodial distribution, calcium influx, and the filopodia-spines transition [15], suggesting that syndecan-2 triggers calcium influx via the CASK-mLIN7-NMDAR complex and induces the filopodia-spines transition (Figure 3).

The morphological feature of the filopodia-spines transition is dendritic spine head enlargement and spine length shortening. The F-actin cytoskeleton must be rearranged to allow for this morphological change. Calcium is known to regulate F-actin dynamics in dendritic spines [54–56], and gelsolin is a calcium-activated F-actin regulator. It acts as an F-actin severing and capping protein [57–59]. Gelsolin deficiency impairs filopodial retraction of developing neurons [60] and inhibits activity-dependent F-actin remodeling in mature dendritic spines [61]. It is also critical for the filopodia-spines transition induced by the syndecan-2-CASK-mLIN7-NMDAR complex, as gelsolin knockdown maintains syndecan-2-induced protrusions at the filopodial stage [15]. It is possible that other calcium regulated F-actin regulators also act downstream of syndecan-2 to control the filopodia-spines transition. More investigations are required to further elucidate the regulation.

### 2.6. Conclusion of the Role of Syndecan-2 Signaling in Dendritic Spine Formation

Through its interactions with intracellular binding partners, the ubiquitously expressing protein syndecan-2 modulates the F-actin cytoskeleton, triggers neurotransmission, and promotes neuron-specific synapse formation. From dendritic filopodia formation, filopodia-spines transition to dendritic spine maturation, syndecan-2 interacts with different binding partners to control F-actin behaviors. Syndecan-2 first activates the PKA pathway via neurofibromin to promote F-actin polymerization and bundling for dendritic filopodia formation [11]. It recruits NMDAR to filopodial tips through its interaction with the CASK-mLIN7 complex and increases the postsynaptic responsiveness to presynaptic stimulation [15]. Calcium influx induces F-actin cytoskeleton rearrangement to allow for the morphological change from filopodia to spines [15]. To further promote dendritic spine maturation and maintenance, syndecan-2 binds to the protein 4.1 through interactions with CASK [16]. Throughout the entire process, neuron specificity falls within NMDAR-mediated calcium influx, which induces F-actin cytoskeleton remodeling to result in morphological changes to the dendritic spine. These studies provide a comprehensive example of how a neuron-specific ion channel coordinates with other adhesion molecules and synaptic proteins to control dendritic spine formation.

## 3. The Neuron-Specific Cytoskeleton Regulator CTTNBP2 Is Highly Associated with Autism Spectrum Disorders

To identify a neuron-specific F-actin regulator involved in dendritic spinogenesis, we searched the database and literature and focused on cortactin-binding protein 2 (CTTNBP2). CTTNBP2 gene encodes a brain-specific protein that interacts with the SH3 domain of cortactin through its proline-rich domain [62]. Cortactin promotes and stabilizes F-actin branching [63, 64] and thus plays a critical role for dendritic spine morphological maintenance [65]. Because cortactin is a ubiquitously expressed protein, its function in controlling dendritic spinogenesis must be regulated by a neuron-specific factor. The specific expression of CTTNBP2 in the brain makes it a good candidate to control cortactin in dendritic spinogenesis. Furthermore,* de novo* mutations in the CTTNBP2 gene have been repeatedly identified in ASD patients [5, 37, 66]. In a genomic analysis covering 3871 ASD patients, results indicated that CTTNBP2 is a high-confidence risk factor for ASDs with a false discovery rate less than 0.05% [5]. These genetic data support a critical role for CTTNBP2 in brain development and function.

### 3.1. CTTNBP2 Variant Transcripts and ASD Mutations

In the expression tag sequence (EST) database (http://www.ncbi.nlm.nih.gov/), three variants have been identified as CTTNBP2 transcripts, namely, CTTNBP2-Short (CTTNBP2-S), CTTNBP2-Intron (CTTNBP2-I), and CTTNBP2-Long (CTTNBP2-L). Based on the nucleotide sequence, the first 625 predicted amino acid residues are shared among all variants [67]. Using an antibody against the common region of the CTTNBP variants, immunoblotting revealed that the Short form of CTTNBP2 is the predominant protein product in brains. The protein products of the Intron and Long forms are undetectable in adult brains [67]. Thus, the following studies of CTTNBP2 in neurons focused on CTTNBP2-S. It is still unclear whether the CTTNBP2-I and CTTNBP2-L variants play any role in neurons. Therefore, mutation analysis of ASD patients is meaningful when the mutation was located within the CTTNBP2-S variant sequences. Seven* de novo* ASD mutations in the CTTNBP2 gene have been identified in the exons encoding CTTNBP2-S [5]. To further explore the association of CTTNBP2 with ASD, these mutations should be investigated in detail to determine their effects on CTTNBP2 molecular function and neuronal morphogenesis.

**Figure 4:**
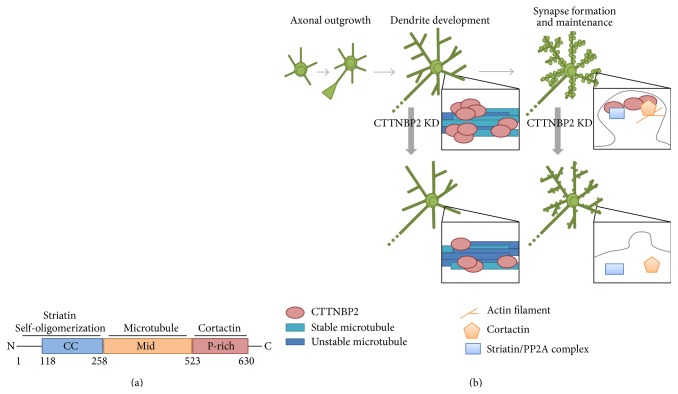
CTTNBP2 and neuronal differentiation. (a) Schematic domain structure and CTTNBP2-S-interacting proteins. CC, coiled-coil domain; Mid, middle region; P-rich, proline-rich domain. (b) The function of CTTNBP2-S in neuronal morphogenesis. CTTNBP2-S controls microtubule stability in the dendritic shafts and cortactin mobility in dendritic spines. Upon CTTNBP2 knockdown during dendritic extension, dendritic complexity decreases. During synaptogenesis, CTTNBP2-S helps maintain cortactin in dendritic spines and promotes dendritic spine formation and maintenance.

Analysis of the amino acid sequence of CTTNBP2-S predicts a coiled-coil domain at the N-terminal region and proline-rich domain at the C-terminus. The middle region does not contain any recognizable protein structure [67]. The N-terminal coiled-coil domain mediates CTTNBP2-S homooligomerization and heterooligomerization of CTTNBP2-S and the striatin family [68, 69]. The C-terminal proline-rich domain interacts with cortactin [62]. The middle region is required for the protein's association with the microtubule cytoskeleton [69]. The functions of these interactions are discussed below (Figure 4).

### 3.2. CTTNBP2-S Controls Cortactin Mobility and Regulates Dendritic Spine Formation and Maintenance

CTTNBP2-S localizes to dendritic spines to control the cortactin-F-actin cytoskeleton. Both endogenous CTTNBP2-S and overexpressed Myc-tagged CTTNBP2-S were found to be highly concentrated at dendritic spines in mature cultured hippocampal neurons. Immunofluorescence analysis of adult brains also indicated that CTTNBP2-S colocalized with F-actin puncta* in vivo*, presumably to dendritic spines [67]. CTTNBP2-S is critical for dendritic spine formation, as CTTNBP2 knockdown right before dendritic spinogenesis at 12 DIV reduces spine density and spine head width measured at 18 DIV. Consistent with the morphological changes, the frequency of mEPSC (miniature excitatory postsynaptic synaptic current) is lower in CTTNBP2 knockdown neurons at 18 DIV [67]. In addition to dendritic spine formation, CTTNBP2-S is involved in dendritic spine maintenance, as CTTNBP2-S knockdown in mature neurons, such as 20 DIV, still reduces dendritic spine density at 26 DIV [67]. Cortactin is required for CTTNBP2-S's regulation of dendritic spinogenesis, as a CTTNBP2-S mutant that cannot interact with cortactin cannot rescue CTTNBP2 knockdown-induced spine deficiency [67]. Moreover, fluorescence recovery after photobleaching (FRAP) analysis indicates that CTTNBP2-S regulates cortactin mobility in mature dendritic spines. In the presence of CTTNBP2-S, cortactin more stably localizes to dendritic spines. The data suggest that CTTNBP2-S retains cortactin in dendritic spines and controls dendritic spine formation and maintenance [67].

CTTNBP2-S also controls distribution of striatin family proteins in dendritic spines [68]. The striatin protein family contains three mammalian members, namely, striatin, zinedin, and SG2NA. They function as B-type regulatory subunits of protein phosphatase 2A (PP2A) to control PP2A subcellular location and substrate specificity [70, 71]. All three striatin family members are highly enriched in dendritic spines [72]. Striatin protein distribution to synapses is mediated by its interactions with CTTNBP2-S through the N-terminal coiled-coil domains of both CTTNBP2-S and striatin family members. Similar to cortactin, CTTNBP2 knockdown impairs dendritic spine targeting of striatins [68]. In conclusion, CTTNBP2-S regulates F-actin dynamics and PP2A signaling at dendritic spines.

### 3.3. CTTNBP2-S Modulates Microtubule Stability and Regulates Dendritic Arborization

In COS cells, exogenous CTTNBP2-S was unexpectedly associated with the microtubule cytoskeleton in addition to the cortactin-F-actin cytoskeleton [69]. Cell-matrix interactions influence the cytoskeleton association of CTTNBP2-S. In COS cells, CTTNBP2-S preferentially associates with the F-actin cytoskeleton within one hour after plating. CTTNBP2-S gradually shifts its preference to the microtubule cytoskeleton when establishing cell-matrix interactions [69]. CTTNBP2-S cytoskeletal associations also change in neurons. CTTNBP2-S is highly concentrated at dendritic spines in mature neurons where CTTNBP2-S interacts with F-actin cytoskeletons. In the premature stages when dendritic spines have not yet formed, CTTNBP2-S is already expressed and forms puncta attached on microtubule bundles along the dendritic shaft [69]. The association of CTTNBP2-S with microtubules increases microtubule stability by bundling the microtubules. Two CTTNBP2-S domains are required for microtubule bundling. The Mid domain is required for the association of microtubule, and the N-terminal coiled-coil domain is involved in CTTNBP2-S oligomerization. Oligomerization allows the CTTNBP2-S oligomer to contain multiple microtubule binding sites to induce microtubule bundling [69]. During the dendritic extension stage, CTTNBP2-S knockdown or disruption of microtubule bundling by overexpression of the N-terminal coiled-coil domain or Mid domain impairs dendritic arborization [69]. The studies suggest that, in addition to controlling the F-actin cytoskeleton, CTTNBP2-S regulates microtubule stability to influence dendrite morphology.

### 3.4. Outstanding Questions about CTTNBP2

The dual roles of CTTNBP2-S in controlling F-actin and the microtubule cytoskeletons require further investigation. As a neuron-specific morphology regulator and a high-confidence risk factor for ASDs, CTTNBP2 deserves further study. Several questions remain to be addressed. For instance, what is the molecular mechanism regulating the association between CTTNBP2-S and F-actin and microtubules? Are the associations of CTTNBP2-S with F-actin and microtubules mutually exclusive? Alternatively, can CTTNBP2-S act as a bridge to link F-actin and microtubules? Only cultured hippocampal neurons have been examined in functional studies of CTTNBP2-S. In the future,* in vivo* studies should be considered. Particularly, to address the association of CTTNBP2 with ASDs, a mouse genetic model is required. The impact of CTTNBP2 ASD mutations on the molecular function of CTTNBP2-S, brain development, and cognition must be studied to further understand the biological significance of CTTNBP2.

## 4. Conclusions

Although hundreds of genes are involved in dendritic spine formation, they should be either neuron-specific or directly or indirectly controlled by or linked to neuron-specific signaling or proteins to specifically regulate dendritic spine formation in neurons. In this review, syndecan-2-induced dendritic spine formation and the role of CTTNBP2-S in controlling neuronal morphology provide two distinct examples of how neuronal morphology can be regulated in a neuron-specific manner. The regulation of neuronal morphology is critical for normal brain function. Understanding these regulations is crucial for basic research and for understanding neurological disorder etiology, which could contribute to potential therapeutic treatments of the diseases.

## Figures and Tables

**Table 1:** SDC2 interacting proteins.

	Binding site in SDC2	Binding site for SDC2	Function
NF1	C1	LRD	Filopodia formation
Ezrin	C1	N-ter.	Links to actin cytoskeleton
Syntenin	C2	PDZ	Cell adhesion and migration
CASK	C2	PDZ	Dendritic spine formation
Synbindin	C2	PDZ-like	Vesicle transport
